# Single-Cell Analysis Reveals Pre-Existing Basal-Associated Epithelial States in Metastatic Hormone-Naïve Prostate Cancer

**DOI:** 10.3390/ijms27125405

**Published:** 2026-06-16

**Authors:** Ryuta Watanabe, Mami Chosei, Tomohisa Sakaue, Noriyoshi Miura, Tadahiko Kikugawa, Takashi Saika

**Affiliations:** 1 Department of Urology, Ehime University Graduate School of Medicine, Toon 791-0295, Japan; miura.noriyoshi.mk@ehime-u.ac.jp (N.M.); takikuga@m.ehime-u.ac.jp (T.K.); saika.takashi.ol@ehime-u.ac.jp (T.S.); 2 Department of Biochemistry and Molecular Genetics, Ehime University Graduate School of Medicine, Toon 791-0295, Japan; chosei.mami.ow@ehime-u.ac.jp; 3 Department of Cardiovascular and Thoracic Surgery, Ehime University Graduate School of Medicine, Toon 791-0295, Japan; sakaue@m.ehime-u.ac.jp

**Keywords:** metastatic hormone-naïve prostate cancer, single-cell transcriptomics, epithelial heterogeneity, basal-associated epithelial states, lineage plasticity, androgen receptor signaling, epithelial–mesenchymal transition

## Abstract

Metastatic hormone-naïve prostate cancer (mHNPC) is a clinically aggressive form of prostate cancer characterized by early systemic dissemination and poor long-term outcomes; however, the intrinsic epithelial cell states present at diagnosis remain poorly defined. In this study, we performed single-cell transcriptomic profiling of diagnostic prostate biopsy specimens from five patients with treatment-naïve mHNPC using Fixed RNA Profiling. Integrated and case-specific analyses characterized epithelial heterogeneity and lineage-associated transcriptional programs. Across 17,825 high-quality single cells, epithelial heterogeneity was identified in all cases. In addition to luminal androgen receptor (AR)-dependent epithelial cells, reproducible basal-associated epithelial populations with reduced AR signaling and stem-like transcriptional features were observed across tumors. Epithelial–mesenchymal transition (EMT)-related transcriptional programs were detected across multiple epithelial states with inter-case variability without forming a distinct EMT cluster, whereas no transcriptionally discrete neuroendocrine epithelial cluster was identified at baseline. These findings demonstrate that treatment-naïve mHNPC harbors pre-existing basal-associated epithelial states that contribute to intrinsic tumor heterogeneity at diagnosis. The presence of AR-low and stem-like epithelial populations prior to systemic therapy suggests a potential role for lineage plasticity in the aggressive biological behavior of metastatic prostate cancer.

## 1. Introduction

Prostate cancer is generally characterized by a relatively indolent clinical course; however, patients who present with metastatic disease at initial diagnosis represent a clinically aggressive subgroup with poor outcomes. The median overall survival of patients with de novo metastatic prostate cancer is approximately five years, highlighting a substantial unmet clinical need [1,2,3].

The initial systemic treatment for metastatic prostate cancer has historically relied on androgen deprivation therapy (ADT). Hormone therapy-naïve metastatic prostate cancer is commonly referred to as metastatic hormone-naïve prostate cancer (mHNPC). Over the past decade, randomized clinical trials have demonstrated that upfront treatment intensification using androgen receptor pathway-targeted agents or docetaxel in combination with ADT improves survival outcomes compared with ADT alone [4,5,6]. These observations suggest that mHNPC may harbor biologically aggressive tumor cell populations that are not fully controlled by hormonal therapy alone.

Despite these advances, the biological basis underlying the aggressive behavior of mHNPC remains incompletely understood. The rapid progression and early metastatic dissemination observed in this disease raise the possibility that substantial intratumoral heterogeneity already exists at diagnosis, prior to systemic treatment. Although genomic and transcriptomic studies have revealed marked heterogeneity in advanced prostate cancer, most prior analyses have relied on bulk sequencing approaches, limiting direct assessment of tumor cell-level diversity [7,8,9].

Single-cell transcriptomic profiling provides a powerful approach to resolve cellular heterogeneity within complex tumor ecosystems. In this study, we performed single-cell transcriptomic analysis of diagnostic prostate biopsy specimens from patients with mHNPC to characterize epithelial tumor cell heterogeneity present at diagnosis. By focusing on treatment-naïve disease, we aimed to delineate early transcriptional states that may contribute to the aggressive clinical behavior of mHNPC.

## 2. Results

### 2.1. Single-Cell Transcriptomic Landscape and Cellular Heterogeneity of mHNPC Biopsy Specimens

**Figure 1:**
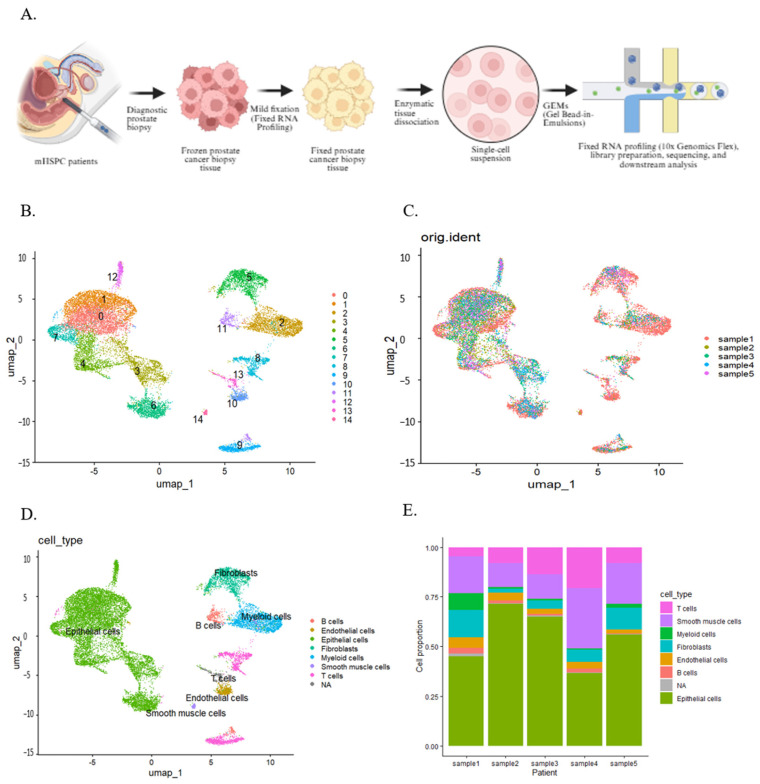
Single-cell transcriptomic landscape of mHNPC. (**A**) Diagnostic prostate biopsy specimens were obtained from patients with mHNPC. Biopsy tissues were cryopreserved and subjected to mild fixation for Fixed RNA Profiling, followed by enzymatic dissociation to generate single-cell suspensions. Single-cell libraries were prepared using the 10× Genomics Fixed RNA Profiling (Flex) platform, sequenced, and analyzed using downstream bioinformatic pipelines. (**B**) UMAP visualization of the integrated single-cell dataset, with cells colored by unsupervised clustering, revealing transcriptionally distinct cell populations. (**C**) UMAP visualization colored by individual patient samples, demonstrating that cells from all cases are broadly distributed across clusters. (**D**) UMAP visualization colored by annotated major cell types based on canonical marker gene expression, including epithelial tumor cells, immune cells (T cells, B cells, and myeloid cells), fibroblasts, endothelial cells, smooth muscle cells, and a minor population of unclassified cells. (**E**) Proportional composition of major cell types across individual mHNPC biopsy samples.

Single-cell transcriptomic profiling was performed on diagnostic prostate biopsy specimens obtained from five patients with mHNPC at the time of initial diagnosis, prior to the initiation of any systemic therapy. As summarized in Figure 1A, prostate biopsy tissues were cryopreserved and subjected to mild fixation for Fixed RNA Profiling, followed by enzymatic dissociation to generate single-cell suspensions. Single-cell libraries were prepared using the 10× Genomics Fixed RNA Profiling (Flex) platform and analyzed using standard downstream bioinformatic pipelines.

Across all samples, sequencing- and cell-level quality control metrics—including the number of recovered cells, sequencing depth, gene detection, and mapping efficiency—are summarized in Supplementary Table S1, Figure S1B. One specimen was processed as a singleplex pilot, whereas the remaining four specimens were processed using multiplexed Fixed RNA Profiling, resulting in expected differences in read depth and mapping efficiency. After applying uniform quality control filtering criteria, a total of 17,825 high-quality single cells were retained for downstream analyses.

Unsupervised analysis of the integrated dataset revealed multiple transcriptionally distinct cell populations, visualized using Uniform Manifold Approximation and Projection (UMAP) (Figure 1B). Coloring by individual patient samples demonstrated that cells from all cases were broadly distributed across clusters, indicating successful integration without strong patient-driven clustering (Figure 1C). Based on canonical marker gene expression, major epithelial, immune, and stromal cell populations were annotated, including epithelial tumor cells, T lymphocytes, B lymphocytes, myeloid cells, fibroblasts, endothelial cells, and smooth muscle cells (Figure 1D; Supplementary Figure S1B,C).

The relative proportions of these major cell populations were largely comparable across cases; however, Case 1 exhibited a lower proportion of epithelial tumor cells compared with the other samples (Figure 1E). These findings indicate that, despite a shared presentation of treatment-naïve metastatic disease, cellular composition varies among individual mHNPC biopsy specimens.

### 2.2. Intra-Epithelial Transcriptional Heterogeneity and Lineage-Associated Programs in mHNPC

**Figure 2:**
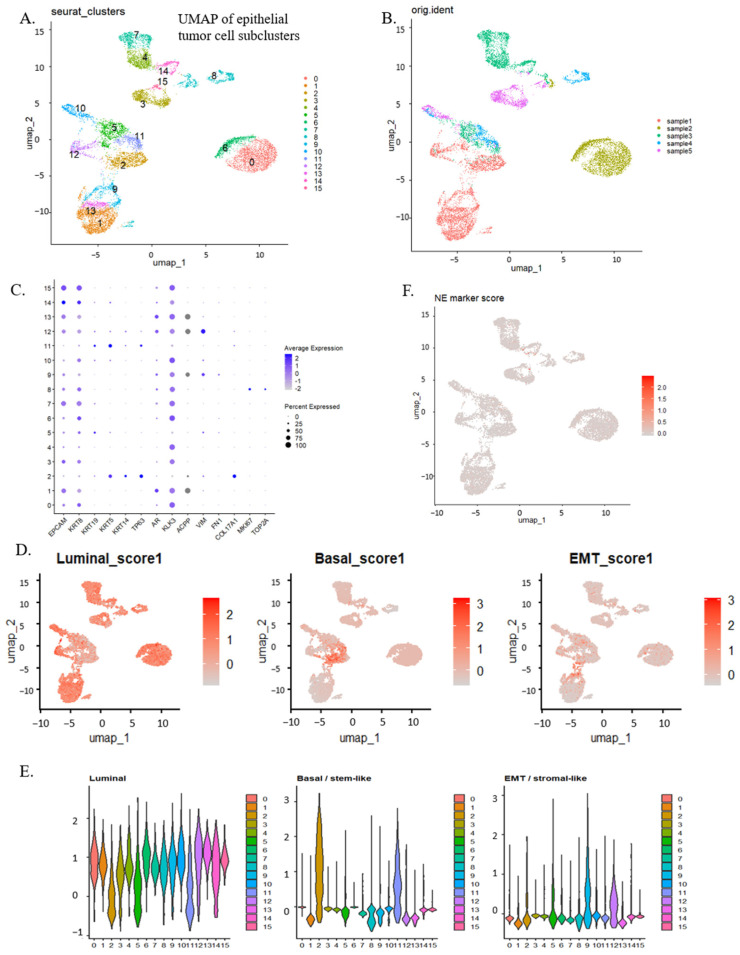
Single-cell transcriptomic landscape of mHNPC. (**A**) UMAP visualization of epithelial tumor cells extracted from the integrated single-cell dataset, colored by unsupervised clustering, showing multiple transcriptionally distinct epithelial subclusters. (**B**) UMAP visualization of epithelial tumor cells colored by individual patient samples, indicating that epithelial subclusters are shared across cases while their relative abundance varies among patients. (**C**) Dot plot showing the expression of representative epithelial marker genes across epithelial subclusters. Dot size represents the percentage of cells expressing each gene, and color intensity indicates the average scaled expression level. (**D**) UMAP feature plots showing lineage-associated transcriptional program scores across epithelial tumor cells, including luminal, basal/stem-like, and EMT/stromal-like programs. (**E**) Violin plots showing the distribution of lineage-associated transcriptional program scores across epithelial subclusters. Luminal-associated programs are broadly distributed, whereas basal/stem-like and EMT/stromal-like programs show relative enrichment in specific subclusters. (**F**) Feature plot showing neuroendocrine marker expression across epithelial tumor cells, demonstrating the absence of a transcriptionally discrete neuroendocrine epithelial cluster at diagnosis.

To characterize epithelial tumor heterogeneity in greater detail, epithelial cells were subsetted from the integrated dataset and re-analyzed independently. In the UMAP embedding of the integrated dataset, epithelial tumor cells formed transcriptionally distinct clusters that were clearly separated from non-epithelial stromal and immune populations (Figure 2A). Subsetting of epithelial cells revealed substantial intra-epithelial heterogeneity, prompting focused re-analysis of this compartment, which was visualized by UMAP (Figure 2B). After re-normalization, principal component analysis was performed, and inspection of the elbow plot identified an inflection point at approximately 30 principal components, which were subsequently used for dimensionality reduction and clustering (Supplementary Figure S2).

Unsupervised re-clustering of epithelial cells identified multiple transcriptionally distinct epithelial subclusters. Representative epithelial marker gene expression across these subclusters is summarized in a dot plot (Figure 2C), confirming marked transcriptional diversity within the epithelial compartment at diagnosis.

To contextualize epithelial subclusters with respect to established prostate cancer lineage programs, lineage-associated transcriptional programs were quantified using predefined gene sets, including luminal/androgen receptor (AR) signaling, basal/stem-like, and EMT/stromal-like programs. UMAP feature plots demonstrated heterogeneous and partially overlapping spatial distributions of these lineage-associated program scores across epithelial tumor cells (Figure 2D). Notably, epithelial clusters enriched for basal-associated transcriptional programs also exhibited elevated stem-like gene set scores, suggesting the presence of a progenitor-like epithelial population within a subset of tumor cells at diagnosis. Violin plots further highlighted distinct enrichment patterns among epithelial subclusters, with luminal-associated programs broadly distributed across multiple subclusters, whereas basal/stem-like and EMT/stromal-like programs showed relative enrichment in specific epithelial subclusters (Figure 2E). In contrast, neuroendocrine marker expression was sparse and did not define a transcriptionally discrete neuroendocrine epithelial cluster at diagnosis (Figure 2F).

### 2.3. Organization of Basal-Associated and EMT/Stromal-like Transcriptional Programs Within Epithelial Tumor Cells

**Figure 3:**
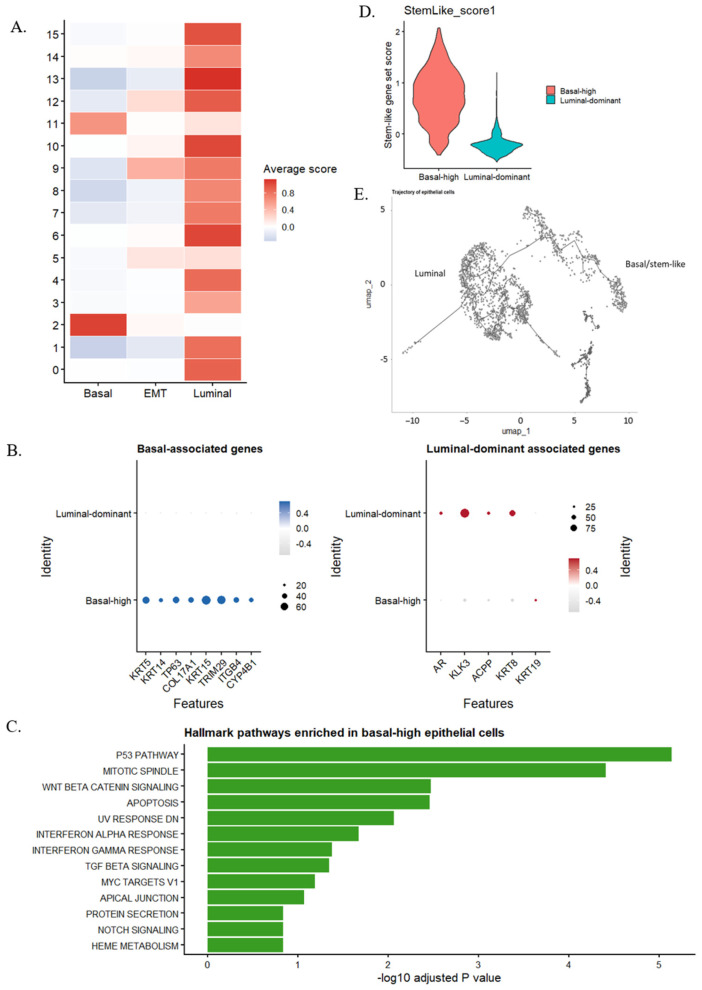
Organization of malignant-associated transcriptional programs within epithelial tumor clusters in mHNPC. (**A**) Heatmap summarizing average lineage-associated transcriptional program scores (luminal, basal/stem-like, and EMT/stromal-like) across epithelial tumor clusters, illustrating the organization of epithelial tumor cells along multiple transcriptional program axes. (**B**) Dot plots showing representative genes enriched in basal-associated and luminal-dominant epithelial tumor clusters. Basal-associated clusters are characterized by enrichment of basal epithelial and undifferentiated markers (e.g., COL17A1, TP63, KRT14, KRT15), whereas luminal-dominant clusters show high expression of canonical luminal and androgen receptor-regulated genes (e.g., AR, KLK3, ACPP, FOLH1). (**C**) Feature plot showing EMT/stromal-like transcriptional program scores across epithelial tumor cells, demonstrating that EMT-related programs are observed across multiple epithelial clusters. (**D**) Box plot showing stem-like gene set scores in basal-associated and luminal-dominant epithelial tumor cells. Basal-associated cells exhibit higher stem-like scores. (**E**) Graph-based trajectory overlaid on the UMAP embedding of epithelial tumor cells, illustrating a continuous epithelial state space spanning luminal-dominant and basal-associated transcriptional programs.

To summarize malignant-associated transcriptional heterogeneity within epithelial tumor cells, lineage-associated transcriptional programs were evaluated at the cluster level. This analysis showed that epithelial tumor cells were organized along multiple transcriptional axes, including luminal-dominant, basal-associated, and EMT/stromal-like programs (Figure 3A).

Basal-associated epithelial clusters (clusters 2 and 11) were characterized by high expression of basal epithelial and undifferentiated markers, including COL17A1, TP63, KRT14, and KRT15. In contrast, luminal-dominant epithelial clusters showed high expression of canonical luminal and androgen receptor-regulated genes such as AR, KLK3, ACPP, and FOLH1, consistent with a differentiated luminal phenotype (Figure 3B).

To assess EMT-related transcriptional features, EMT/stromal-like transcriptional programs were quantified using a predefined gene set. Importantly, the EMT/stromal-like program was observed across multiple epithelial clusters, including both basal-associated and non-basal clusters, indicating that EMT-related transcriptional features are not confined to a single epithelial cluster but represent a program that spans distinct epithelial states (Figure 3C).

Basal-associated epithelial tumor cells exhibited higher stem-like gene set scores compared with luminal-dominant epithelial tumor cells (Figure 3D). The stem-like gene set comprised genes associated with progenitor and self-renewal properties, including SOX2, KLF4, ITGA6, PROM1, and ALDH1A1, supporting a less differentiated transcriptional state within basal-associated clusters.

Graph-based trajectory analysis further demonstrated that luminal-dominant and basal-associated transcriptional states were connected through a continuous epithelial state space without discrete boundaries (Figure 3E), consistent with transcriptional continuity across epithelial tumor cell states.

### 2.4. Case-Specific and Integrated UMAP Analyses of Basal-Associated and EMT-Related States in mHNPC

**Figure 4:**
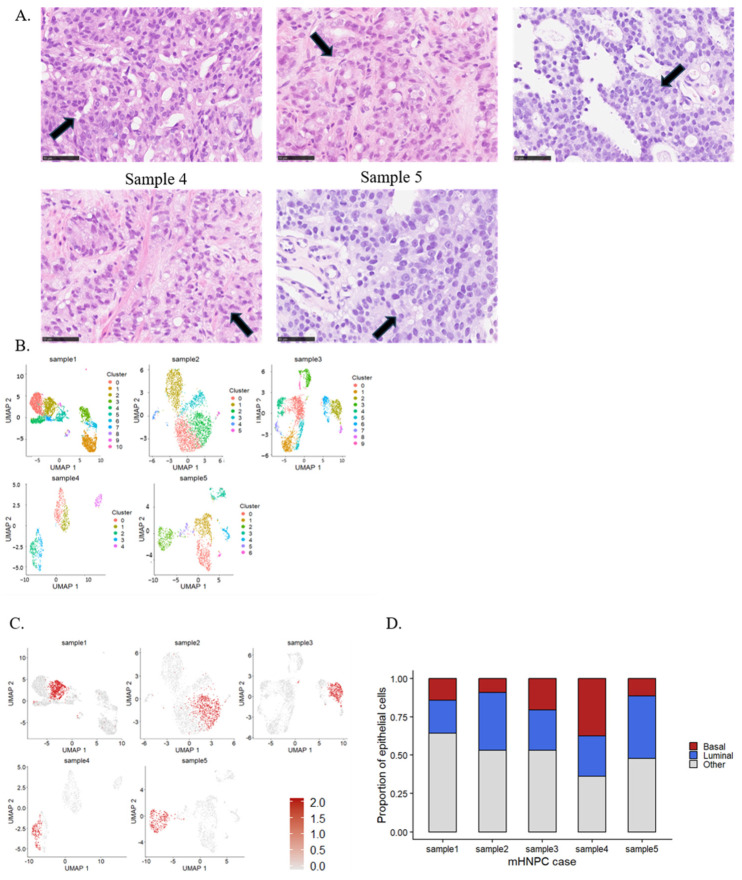
Case-specific and integrated UMAP analyses of basal-associated and EMT-related transcriptional states in mHNPC. (**A**) Representative hematoxylin and eosin (H&E) staining of diagnostic prostate biopsy specimens from the five mHNPC cases. Tumor glands composed of malignant epithelial cells are indicated by arrows. The tumor cells show enlarged nuclei with prominent nucleoli and form irregular glandular structures consistent with prostatic adenocarcinoma. Scale bars = 200 μm. (**B**) Case-specific UMAP embeddings of epithelial tumor cells generated independently for each mHNPC case using identical preprocessing and dimensionality reduction parameters. (**C**) Basal-associated transcriptional states highlighted on case-specific UMAPs, demonstrating their presence across all cases with variable spatial distribution. (**D**) Stacked bar plots showing the proportional composition of epithelial tumor cell clusters across individual mHNPC cases. Epithelial tumor cells were classified into basal-associated (clusters 4 and 5), luminal-dominant (clusters 2, 7, 9, 11, 12, and 13), and other clusters based on marker gene expression. Percentages shown above each bar indicate the fraction of basal-associated epithelial cells relative to all cells in each case.

Representative histopathological features of diagnostic prostate biopsy specimens from the five mHNPC cases are shown in Figure 4A.

To examine the case-level organization of epithelial transcriptional states, UMAP embeddings were generated independently for each mHNPC case using identical preprocessing and dimensionality reduction parameters. These case-specific UMAPs revealed comparable epithelial transcriptional landscapes across individual tumors, while preserving case-specific spatial distributions of epithelial clusters (Figure 4B).

Basal-associated transcriptional states were detected in all individual tumors, although their spatial distribution varied among cases (Figure 4C). In each case-specific UMAP, basal-associated cells were distributed within the epithelial landscape rather than forming completely isolated populations.

To quantify inter-case variability in epithelial cluster composition, the proportional distribution of epithelial clusters was examined across the five mHNPC cases (Figure 4D). Epithelial cells were classified into basal-associated clusters (clusters 4 and 5), luminal-dominant clusters (clusters 2, 7, 9, 11, 12, and 13), and other clusters based on marker gene expression. While luminal-dominant clusters comprised the majority of epithelial tumor cells in all cases, the fraction of basal-associated epithelial cells varied among tumors.

To assess whether these transcriptional states were organized within a shared epithelial landscape across tumors, epithelial tumor cells from individual cases were projected onto the integrated UMAP derived from the combined analysis of all samples (Supplementary Figure S3A). Basal-associated transcriptional states were consistently observed across cases within the integrated epithelial landscape (Supplementary Figure S3B), whereas EMT-related transcriptional program scores exhibited greater variability among tumors (Supplementary Figure S3C).

Collectively, these analyses indicate that basal-associated transcriptional states represent a recurrent epithelial feature across mHNPC tumors, while the distribution of epithelial clusters and EMT-related programs varies among individual cases.

## 3. Discussion

In this study, single-cell transcriptomic profiling of diagnostic prostate biopsy specimens revealed that substantial epithelial heterogeneity is already present at the time of diagnosis in mHNPC. While pronounced intratumoral heterogeneity has been extensively documented in CRPC, particularly in the context of lineage plasticity and treatment resistance, single-cell analyses focusing on treatment-naïve metastatic disease remain limited. Our findings indicate that epithelial transcriptional heterogeneity is not merely a consequence of therapeutic selection but is an intrinsic feature of mHNPC prior to systemic treatment [10].

A central observation of this study is the consistent detection of basal-associated epithelial states across all analyzed cases. These basal-associated tumor cells were characterized by reduced AR-related transcriptional activity, enrichment of basal epithelial markers, and elevated stem-like gene set scores, reflecting a less differentiated epithelial state. Basal or stem-like transcriptional programs have been predominantly described in treatment-resistant settings, including CRPC and aggressive variant prostate cancer, where they have been linked to lineage plasticity and poor response to AR-targeted therapies [11,12,13,14,15]. Our data extend these observations by demonstrating that such transcriptional states are already detectable at diagnosis in mHNPC, rather than being exclusively acquired under therapeutic pressure. Consistent with this notion, experimental lineage-tracing studies have shown that prostate cancer can originate from basal cells and subsequently be maintained by AR-low luminal-like tumor cells, underscoring intrinsic lineage plasticity within prostate epithelial compartments [14].

In addition to basal-associated states, we identified epithelial tumor cells exhibiting EMT-related transcriptional signatures. This observation is consistent with previous reports describing transcriptomic heterogeneity in metastatic hormone-sensitive prostate cancer [16]. EMT-related features did not define a discrete epithelial cluster but were observed as a transcriptional program spanning multiple epithelial clusters, including both basal-associated and non-basal populations, with variable enrichment across cases. This observation is consistent with prior studies indicating that EMT represents a dynamic and context-dependent transcriptional program rather than a fixed differentiation state [17,18,19,20]. Thus, the heterogeneous activation of EMT-related programs at diagnosis likely reflects epithelial plasticity rather than uniform acquisition of a mesenchymal phenotype. Importantly, our findings do not support the presence of a fully established mesenchymal state at diagnosis. Rather, EMT-related transcriptional programs appear to represent partial or intermediate states of epithelial plasticity distributed across multiple epithelial populations.

Recent single-cell studies of metastatic castration-sensitive prostate cancer have primarily focused on treatment-associated changes in tumor and immune cell populations. In particular, Hawley and colleagues demonstrated that tumor cell states dynamically shift in response to systemic therapy, highlighting state plasticity during treatment [21]. In contrast, the present study delineates the epithelial transcriptional landscape present at diagnosis, prior to systemic therapy, indicating that basal-associated and EMT-related programs are already embedded before treatment-induced selection. Trajectory-based analyses further support this interpretation by suggesting that luminal-dominant and basal-associated epithelial states occupy a shared epithelial state space and are not completely discrete populations. However, these analyses did not indicate a clear hierarchical differentiation process or directional transition between the two states [10,22].

Given prior experimental evidence that potent androgen pathway suppression can induce lineage plasticity toward neuroendocrine prostate cancer, often through intermediate basal-like or stem-like states [12,13,15], we examined the expression of canonical neuroendocrine markers in our diagnostic mHNPC samples. However, we did not observe clear or consistent upregulation of neuroendocrine-associated genes at diagnosis. This finding indicates that overt activation of a neuroendocrine transcriptional program is not a defining feature of mHNPC at baseline. Rather, these results are compatible with a model in which basal-associated epithelial cells represent a plastic state that may acquire neuroendocrine features only under additional selective pressures, such as sustained AR pathway inhibition during subsequent therapy, as suggested by prior molecular and clinical studies [12,13,15,23,24,25,26]. Importantly, canonical neuroendocrine transcriptional programs were not enriched in basal-associated epithelial cells, and no transcriptionally distinct neuroendocrine epithelial cluster was identified at diagnosis. Therefore, the basal-associated states identified in this study should be interpreted as epithelial plasticity-associated states rather than early neuroendocrine prostate cancer (NEPC) states.

Several molecular alterations have been implicated in epithelial plasticity in prostate cancer. At the molecular level, reduced PTEN expression has been associated with diminished AR signaling, enrichment of EMT- and lineage plasticity-related signatures, and poor clinical outcomes in metastatic hormone-sensitive prostate cancer [27]. Although direct genotype–phenotype relationships could not be addressed in the present study, as the Flex platform is based on probe-derived transcript detection and does not provide mutation-level information, such molecular contexts may contribute to the transcriptional states observed at diagnosis in mHNPC.

Despite these findings, several limitations should be acknowledged. First, the sample size was limited to five patients, and therefore the findings should be considered hypothesis-generating and require validation in larger independent cohorts. However, treatment-naïve mHNPC specimens suitable for single-cell transcriptomic analysis remain relatively uncommon, and the present cohort represents a valuable clinical resource for investigating the intrinsic epithelial states present at diagnosis. Second, although whole-transcriptome probe panels were used, the Flex platform relies on probe-based transcript detection and therefore does not provide information on sequence variants or full-length transcript structures. Future studies integrating larger multicenter cohorts, broader transcriptomic platforms, and longitudinal sampling before and after systemic therapy will be important to further clarify the clinical significance of basal-associated epithelial states and their relationship to treatment resistance and lineage plasticity.

In summary, our single-cell analysis reveals that mHNPC tumors exhibit substantial epithelial heterogeneity at diagnosis, characterized by the coexistence of luminal-dominant, basal-associated, and EMT-related transcriptional programs. Basal-associated states represent a shared and reproducible feature of mHNPC, whereas EMT-related programs display inter-case variability. These observations extend existing conceptual frameworks of lineage plasticity and treatment-resistant prostate cancer discussed in recent reviews [15,28,29,30], and provide biological context for the aggressive clinical behavior of treatment-naïve metastatic prostate cancer.

## 4. Materials and Methods

### 4.1. Patient Samples and Clinical Information

Patients who underwent transrectal prostate biopsy at Ehime University Hospital between November 2021 and June 2024 and were subsequently diagnosed with metastatic hormone-naïve prostate cancer (mHNPC) were eligible for inclusion in this study. Diagnostic biopsy specimens obtained prior to the initiation of any systemic therapy were subjected to single-cell transcriptomic analysis. All patients provided written informed consent prior to sample collection. This study was conducted in accordance with the Declaration of Helsinki and was approved by the institutional review board of Ehime University Hospital (IRB approval number: 2211017). Clinical and pathological characteristics of the patients, including age, serum prostate-specific antigen levels, tumor stage, metastatic status, metastatic sites, and disease volume, are summarized in Table 1.

All patients were diagnosed with mHNPC and underwent prostate biopsy prior to the initiation of any systemic therapy. Clinical staging was determined at diagnosis according to the TNM classification. Pathological diagnosis and Gleason grading were based on prostate biopsy specimens. PSA denotes prostate-specific antigen.

Disease volume was classified according to the CHAARTED criteria; high-volume disease was defined as the presence of visceral metastases or ≥4 bone metastases with at least one lesion beyond the vertebral bodies and pelvis. All biopsy specimens analyzed in this study were obtained prior to the initiation of any systemic therapy. Following diagnostic biopsy and sample collection, Case 1 received upfront enzalutamide plus ADT, Cases 2–4 received ADT alone, and Case 5 received triplet therapy consisting of ADT, docetaxel, and darolutamide.

### 4.2. Single-Cell Dissociation and Library Preparation

Fresh biopsy specimens were enzymatically dissociated into single-cell suspensions using collagenase-based digestion, followed by mechanical trituration and filtration to remove debris. Cell viability was assessed prior to downstream processing. Single-cell RNA sequencing libraries were generated using the Chromium Single Cell Fixed RNA Profiling (Flex) platform (10× Genomics, Pleasanton, CA, USA) according to the manufacturer’s protocol. This platform enables single-cell transcriptomic analysis of fixed specimens through probe-based transcript capture following mild fixation and cell dissociation. Whole-transcriptome probe panels were used, allowing for simultaneous evaluation of epithelial, immune, and stromal cell populations rather than focusing on a tumor-specific targeted gene panel. Libraries were sequenced on an Illumina platform (Illumina, San Diego, CA, USA).

### 4.3. Preprocessing, Quality Control, and Data Integration

Raw sequencing data were processed using Cell Ranger (v7.1.0; 10× Genomics, Pleasanton, CA, USA) for read alignment, barcode demultiplexing, and unique molecular identifier (UMI) counting. Downstream analyses were performed using Seurat (v5.4.0) in R (v4.5.1). Cells were filtered to exclude low-quality cells and potential doublets using the following criteria: number of detected genes per cell (nFeature_RNA) >200 and <6000, and proportion of mitochondrial gene expression (percent.mt) <15%. The same quality control thresholds were uniformly applied across all samples.

Data normalization and variance stabilization were performed using SCTransform (sctransform v0.4.3). To integrate data across patients and mitigate batch effects, Harmony-based integration was applied using Harmony (v1.2.4) with patient identity as the batch variable. Principal component analysis (PCA) was performed on the integrated dataset, followed by construction of a shared nearest neighbor graph and unsupervised clustering using the Louvain algorithm. Dimensionality reduction was visualized using Uniform Manifold Approximation and Projection (UMAP).

### 4.4. Cell Type Annotation and Epithelial Subclustering

Major cell types were annotated based on canonical marker gene expression, including epithelial, immune, and stromal populations. Epithelial tumor cells were subsequently subsetted from the integrated dataset and re-analyzed independently to characterize tumor cell-intrinsic heterogeneity. After re-normalization, PCA was performed and the first 30 principal components were selected for dimensionality reduction, clustering, and UMAP visualization of epithelial subclusters.

### 4.5. Differential Expression and Lineage-Associated Program Analysis

Differentially expressed genes for epithelial subclusters were identified using the Wilcoxon rank-sum test implemented in Seurat, with adjustment for multiple testing. Lineage-associated transcriptional programs, including luminal/androgen receptor, basal/stem-like, neuroendocrine-like, and epithelial–mesenchymal transition-related signatures, were quantified using predefined gene sets and module scoring. Program scores were visualized on UMAP embeddings and compared across epithelial subclusters.

### 4.6. Statistical Analysis

All statistical analyses and data visualization were performed in R (v4.5.1) using Seurat (v5.4.0), ggplot2, and patchwork. Statistical significance was defined as an adjusted *p* value < 0.05. Detailed experimental procedures and bioinformatic analyses are provided in the Supplementary Materials.

## Figures and Tables

**Table 1:** Baseline clinical and pathological characteristics of the five patients with treatment-naïve mHNPC.

Patient	Age	PSA at Diagnosis (ng/mL)	Clinical TNM Stage	CHAARTED Volume	Gleason Score
Case 1	76	451	cTx N1 M1b (Th12)	Low	4 + 5 = 9
Case 2	74	256.4	cT3b N1 M1a (para-aortic LN)	Low	5 + 4 = 9
Case 3	54	694.5	cT3a N1 M1c (Lung)	High	4 + 4 = 8
Case 4	75	2300	cT3 N1 M1b (ribs, vertebrae, scapula, and pelvis)	High	4 + 5 = 9
Case 5	44	106	cT4 N1 M1b (Sacral bone metastasis, obturator and para-aortic lymph nodes)	Low	4 + 5 = 9

## Data Availability

The RNA sequencing data generated in this study have been deposited in the Gene Expression Omnibus (GEO) repository under accession number GSE318581 (https://www.ncbi.nlm.nih.gov/geo/query/acc.cgi?acc=GSE318581, accessed on 22 May 2026).

## Supplementary Materials

The following supporting information can be downloaded at: https://www.mdpi.com/article/10.3390/ijms27125405/s1.

## Abbreviations

The following abbreviations are used in this manuscript:

ADT	androgen deprivation therapy
AR	androgen receptor
AVPC	aggressive variant prostate cancer
CRPC	castration-resistant prostate cancer
DEG	differentially expressed gene
EMT	epithelial–mesenchymal transition
Flex	Fixed RNA Profiling
GEO	Gene Expression Omnibus
mHNPC	metastatic hormone-naïve prostate cancer
NEPC	neuroendocrine prostate cancer
PCA	principal component analysis
PSA	prostate-specific antigen
QC	quality control
UMAP	Uniform Manifold Approximation and Projection
UMI	unique molecular identifier
