# Supplementary material for: Single-Cell Analysis Reveals Pre-Existing Basal-Associated Epithelial States in Metastatic Hormone-Naïve Prostate Cancer
Source: Int J Mol Sci. 2026 Jun 16;27(12):5405. doi: 10.3390/ijms27125405 (PMC13299256; doi:10.3390/ijms27125405)

Supplementary Figure S1. Quality control and cell type annotation of single-cell Fixed RNA Profiling data.

(A) Violin plots showing distributions of quality control metrics after filtering.

(B) Dot plot showing expression of canonical marker genes used for cell type annotation across clusters.

(C) Feature plots showing representative marker gene expression overlaid on the UMAP embedding.

Supplementary Figure S2. Selection of principal components for epithelial tumor cell analysis.

Elbow plot showing variance explained by principal components derived from epithelial tumor cells following re-normalization. The first 30 principal components were selected for downstream analyses.

Supplementary Figure S3. Case-wise projection onto the integrated epithelial UMAP.

(A) Integrated UMAP embedding of epithelial tumor cells, with cells from individual mHNPC cases highlighted, demonstrating that epithelial subclusters are shared across cases.

(B) Projection of basal-associated transcriptional states onto the integrated UMAP, showing their distribution across multiple cases.

(C) Projection of EMT-related transcriptional program scores onto the integrated UMAP, illustrating inter-case variability in EMT-related features.

Supplementary Table S1. Quality control metrics of single-cell Fixed RNA Profiling datasets.

Summary of sequencing- and cell-level quality control metrics for five mHNPC biopsy specimens analyzed by Fixed RNA Profiling.

Supplement Table S1

Sample	Cells recovered	Mean reads per cell	Median genes per cell	Median UMI counts per cell	Total genes detected	Confidently mapped reads (%)	Notes
Case 1	8,864	5,949	1,263	1,816	17,431	85.02	Singleplexed
Case 2	3,106	23,859	975	1,475	15,626	66.52	Multiplexed
Case 3	2,947	34,816	749	1,108	17,978	56.37	Multiplexed
Case 4	1,124	25,813	860	1,110	15,704	54.56	Multiplexed
Case 5	1,784	43,232	1,026	1,529	16,942	61.86	Multiplexed

Supplement Figure S1A

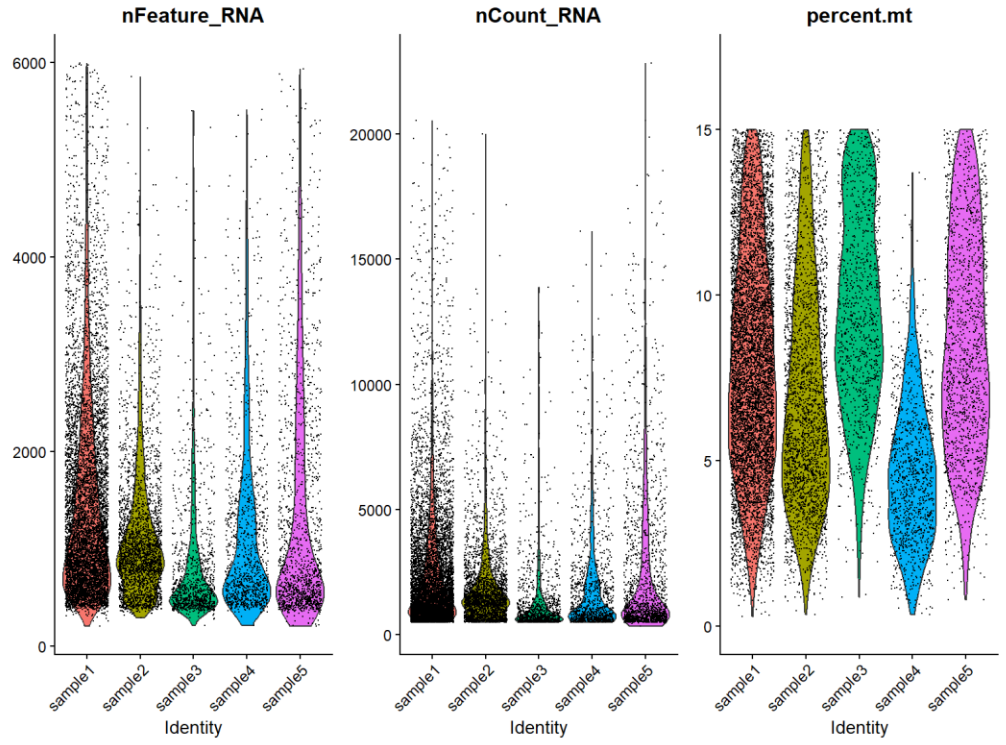

Supplement Figure S1B

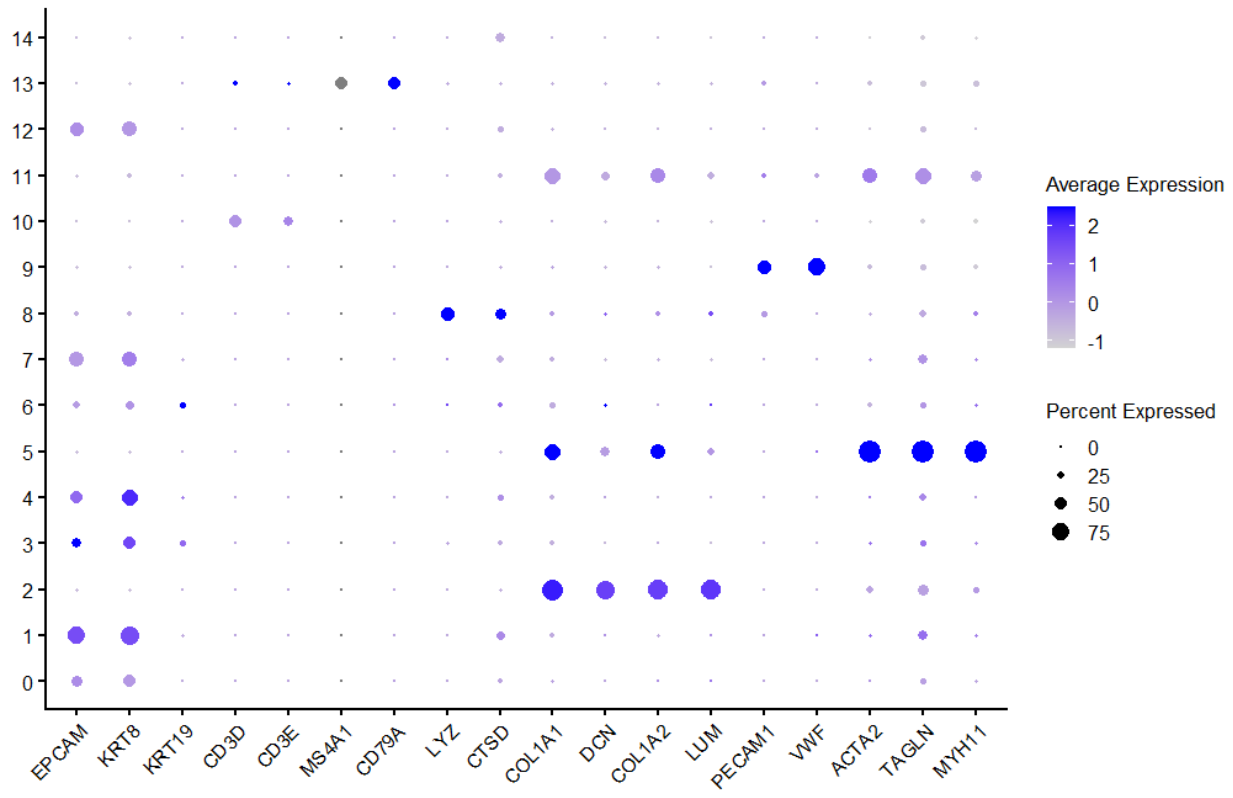

Supplement Figure S1C

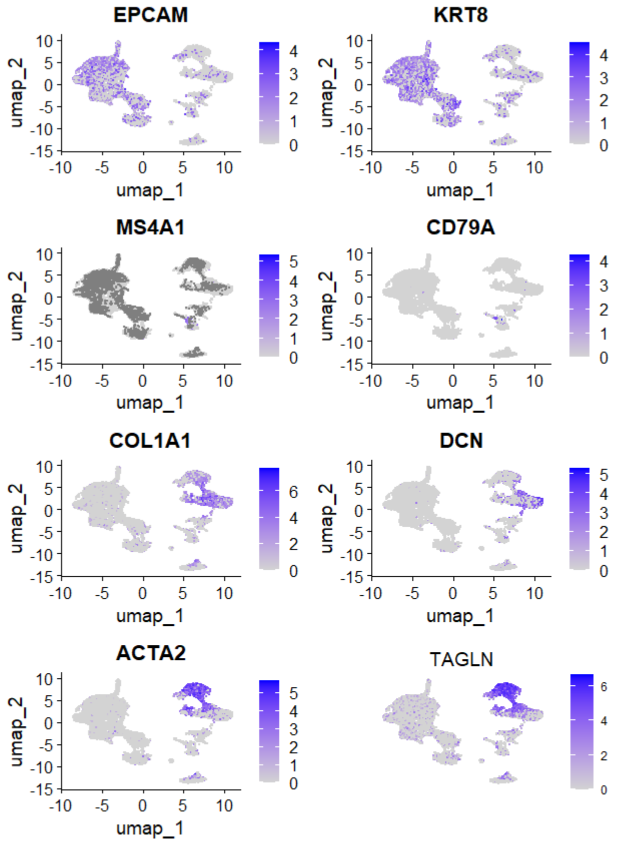

Supplement Figure S2

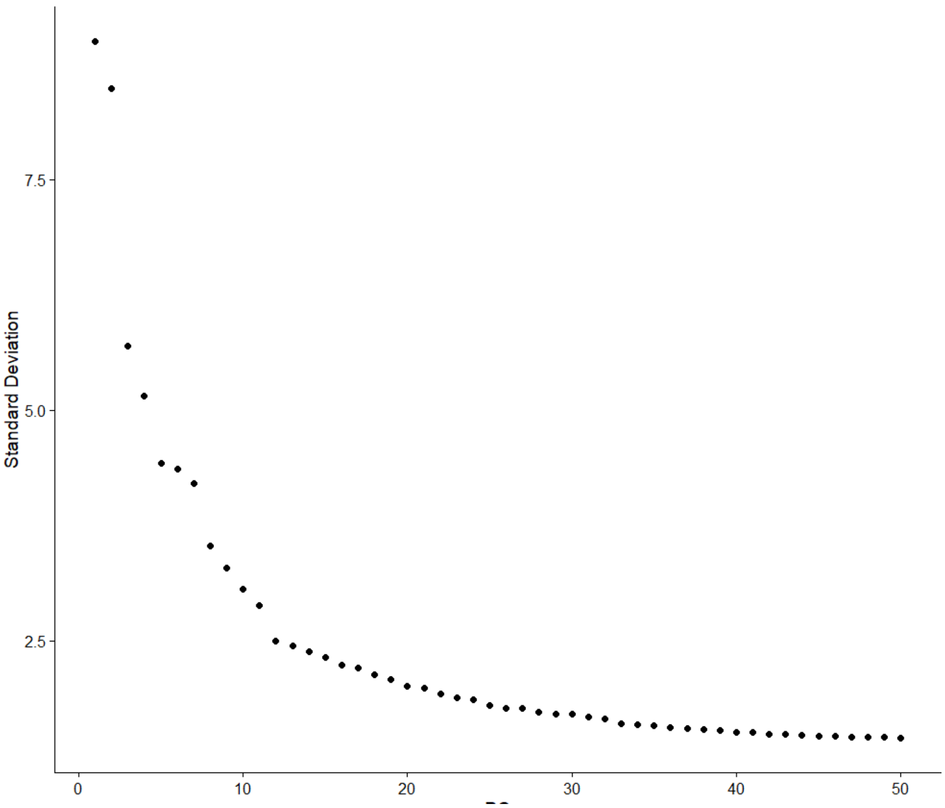

Supplement Figure S3A

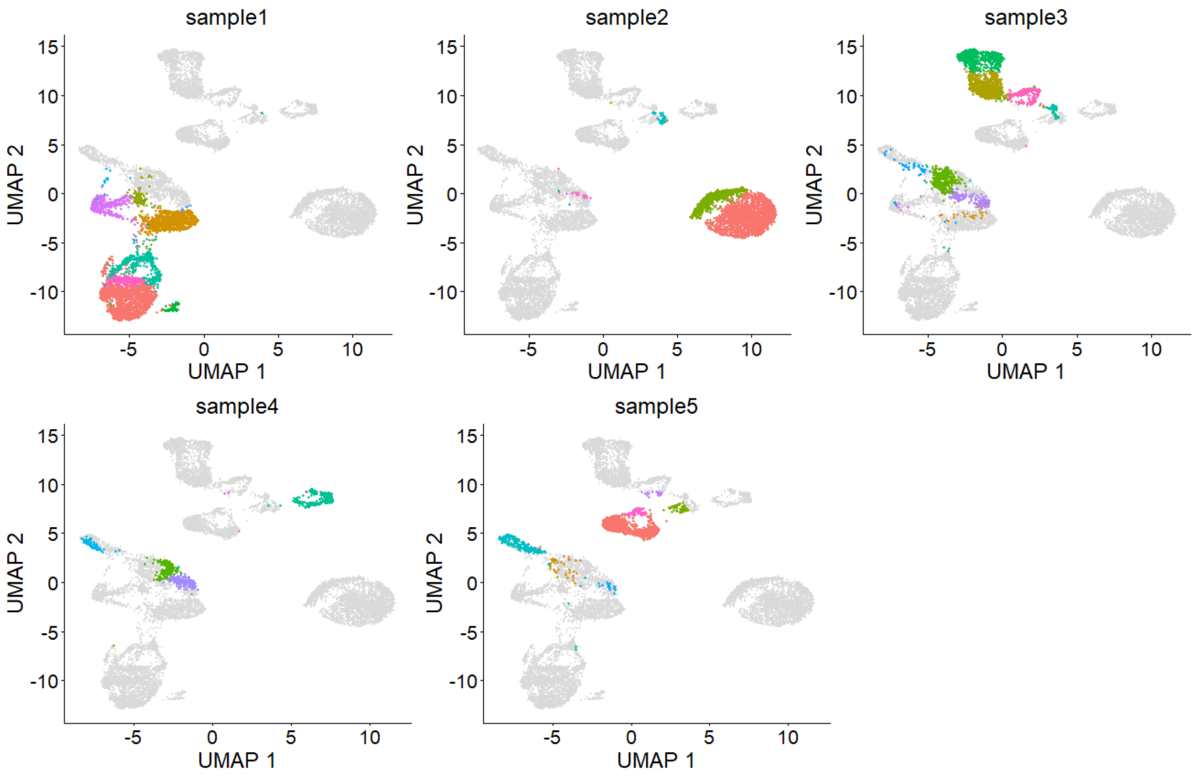

Supplement Figure S3B

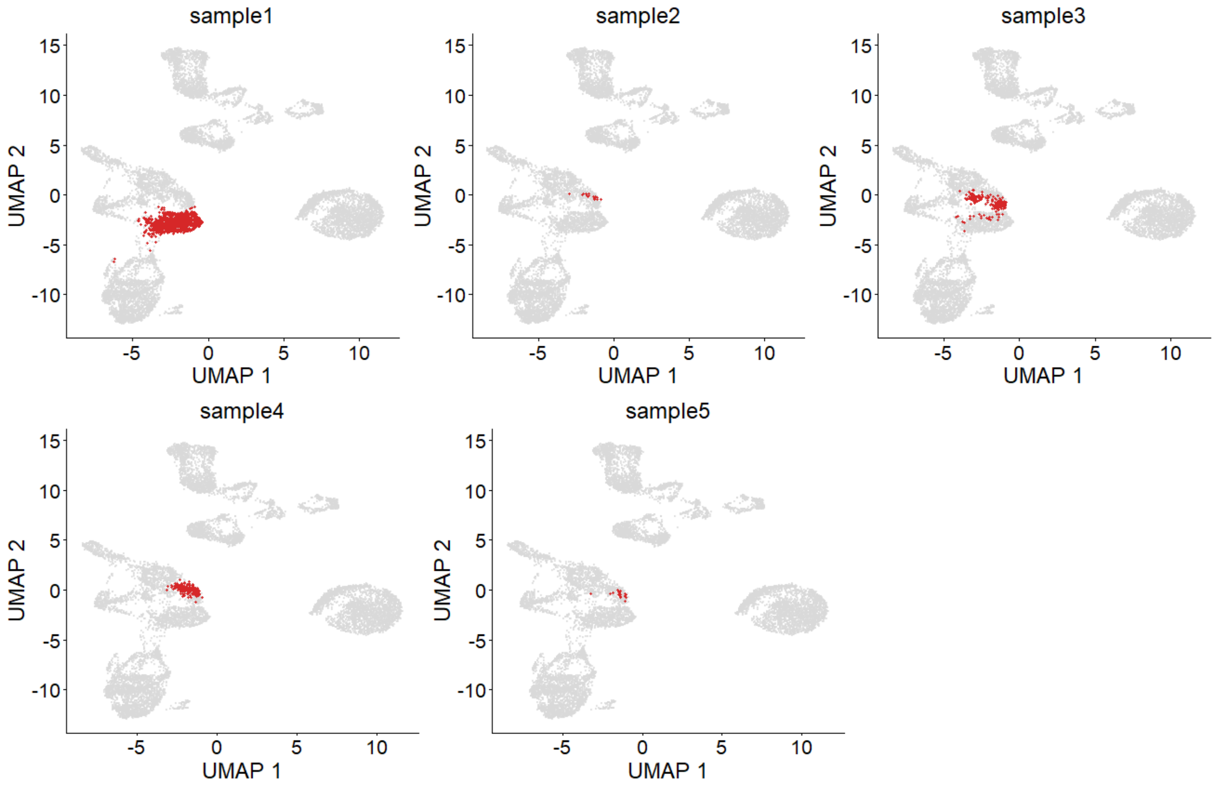

Supplement Figure S3C

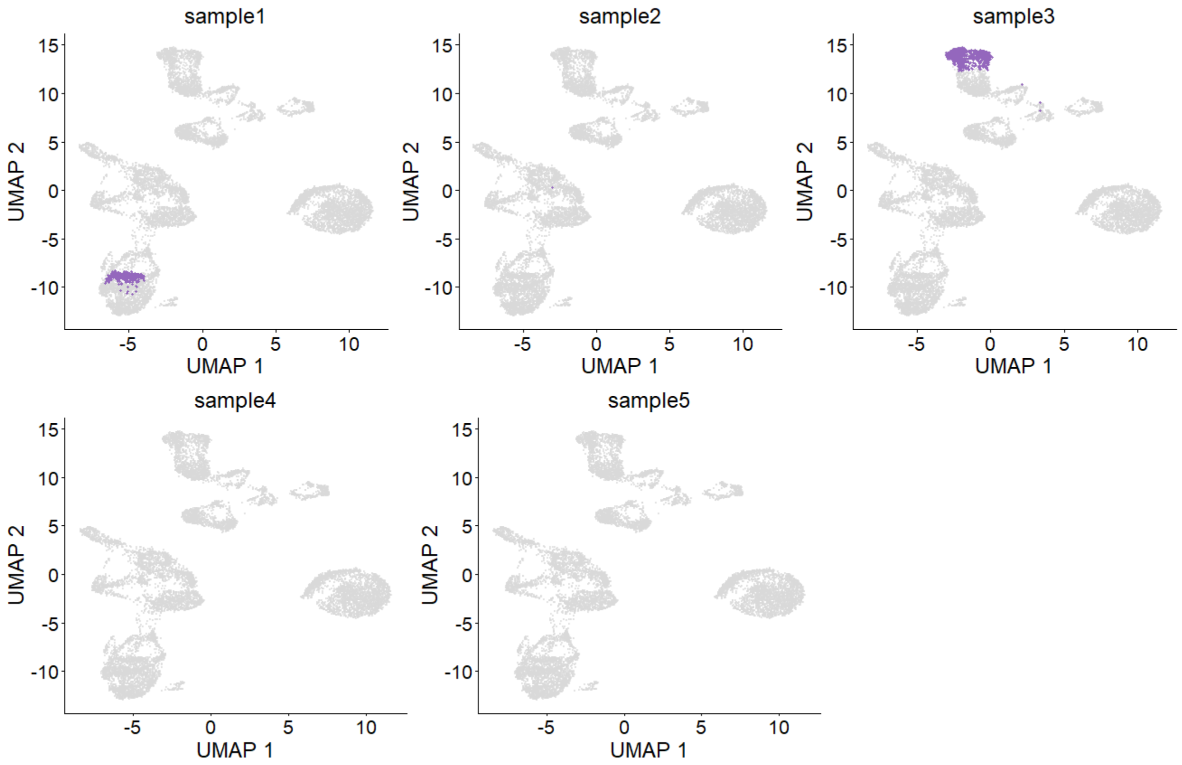

Supplement: Supplementary file 1 [file ijms-27-05405-s001.zip › ijms-4364996-supplementary.pdf]
